# Preparation of Composite Materials from Self-Assembled Chitin Nanofibers

**DOI:** 10.3390/polym13203548

**Published:** 2021-10-14

**Authors:** Jun-ichi Kadokawa

**Affiliations:** Graduate School of Science and Engineering, Kagoshima University, 1-21-40 Korimoto, Kagoshima 890-0065, Japan; kadokawa@eng.kagoshima-u.ac.jp; Tel.: +81-99-285-7743

**Keywords:** bottom-up, chitin nanofibers, composite materials, deep eutectic solvents, ionic liquids, nanochitins, regeneration, self-assembling

## Abstract

Although chitin is a representative abundant polysaccharide, it is mostly unutilized as a material source because of its poor solubility and processability. Certain specific properties, such as biodegradability, biocompatibility, and renewability, make nanofibrillation an efficient approach for providing chitin-based functional nanomaterials. The composition of nanochitins with other polymeric components has been efficiently conducted at the nanoscale to fabricate nanostructured composite materials. Disentanglement of chitin microfibrils in natural sources upon the top-down approach and regeneration from the chitin solutions/gels with appropriate media, such as hexafluoro-2-propanol, LiCl/*N, N*-dimethylacetamide, and ionic liquids, have, according to the self-assembling bottom-up process, been representatively conducted to fabricate nanochitins. Compared with the former approach, the latter one has emerged only in the last one-and-a-half decade. This short review article presents the preparation of composite materials from the self-assembled chitin nanofibers combined with other polymeric substrates through regenerative processes based on the bottom-up approach.

## 1. Introduction

**Figure 1:**
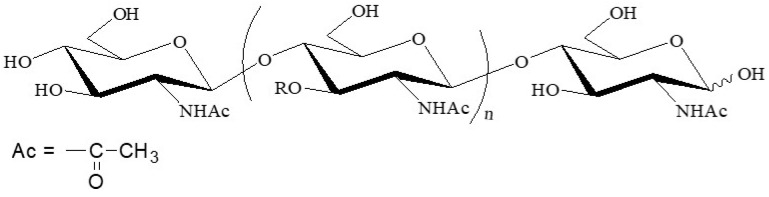
Chemical structure of chitin.

The conversion of biomasses into value-added materials as alternatives to petroleum-based conventional materials has increasingly attracted much attention based on views revolving around the environmental aspects of the earth [1]. Chitin is one of the most abundant polysaccharides comparable to cellulose and, accordingly, has been expected to be used as a biomass source in bio-based practical materials. It is an aminopolysaccharide consisting of repeating β(1→4)-linked *N*-acetyl-D-glucosamine units (Figure 1), which occurs mainly in the exoskeletons of crustacean shells, such as crab and shrimp shells, in nature [2,3,4]. However, chitin is mostly unutilized in practical applications even today, principally because of its poor solubility and processability owing highly to crystallinity and stiff polymeric chain packing by numerous hydrogen bonds. Therefore, research regarding the conversion of chitin into value-added bio-based materials has continued diligently in recent years [5]. Nanofibrillation is one of the most efficient approaches for the functionalization of chitin, which involves the fabrication of nanocrystals and nanofibers (nanochitins) because of the remarkable properties of bio-based nanomaterials, such as their lightweight character, high tensile strength, low thermal expansion coefficient, biocompatibility, and nanosheet formability for sensing and electronic devices [6,7,8,9,10,11,12,13]. There are two practical processes to fabricate nanofibrillated chitins from native chitin sources, consisting of disentanglement of chitin microfibrils upon the top-down approach and regeneration from the chitin solutions/gels with appropriate media according to the self-assembling bottom-up process [14].

**Figure 2:**
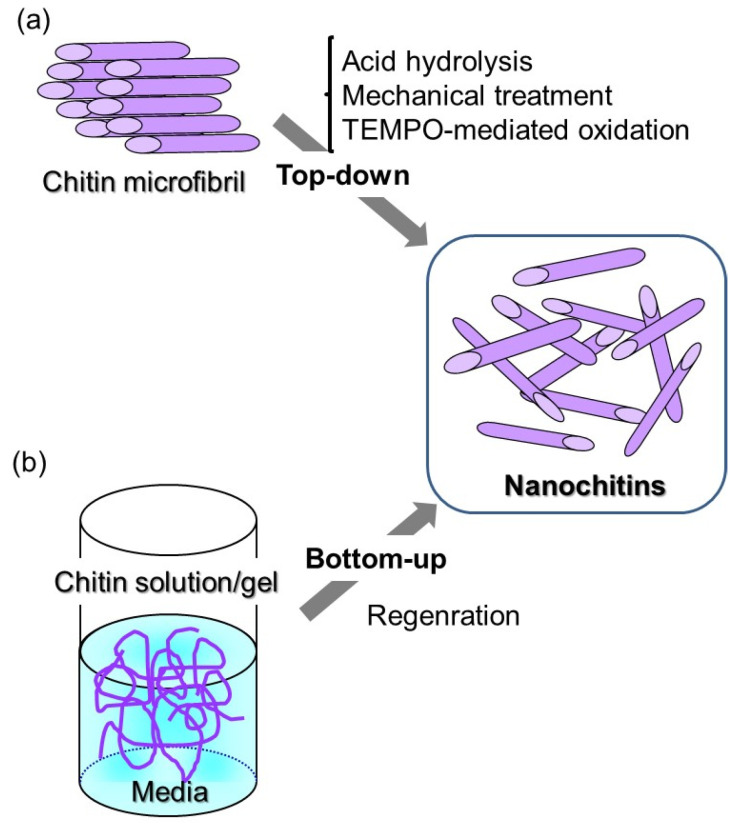
Fabrication of nanochitins upon: (**a**) top-down; and (**b**) bottom-up approaches.

Native chitin in crustacean shells is arranged as microfibrils embedded in a protein matrix, which comprises nanofibers with 2–5 nm diameters and extended fibrous crystalline structures [15,16]. Accordingly, some top-down techniques, including acid hydrolysis [17], mechanical treatment by grinding technique [18,19,20,21], and 2,2,6,6-tetramethylpiperidine-1-oxyl radical (TEMPO)-mediated oxidation [22,23,24], have been developed to fabricate nanochitins with different morphologies and sizes (Figure 2a). The resulting chitin nanofibers (ChNFs) are currently used as additives to mix with cosmetics and biomedical materials for increasing the moisture and water content of skin and suppressing inflammation from skin damage.

Compared with the above top-down approach, bottom-up techniques for the fabrication of nanochitins have only emerged in the last one-and-a-half decade [25,26]. Such techniques have mainly been achieved by the simple regeneration from chitin solutions/gels, resulting in self-assembled ChNFs (Figure 2b). The composition of resulting ChNFs with other polymeric components at the nanoscale has been further conducted to fabricate ChNF-based nanocomposite materials for improving properties and providing new functions [27]. This short review article presents an efficient approach for the preparation of the nanostructured composite materials, ranging from the self-assembled ChNFs combined with other polymeric components, to the facile regenerative processes based on the bottom-up approach, where CNFs and other constituent materials are merged to create new materials with properties different from the individual components. The bottom-up process for the fabrications of composite materials has an advantage regarding easy preparation compared with the top-down process, because the former technique does not generally require any special equipment. Therefore, the regenerative bottom-up method will contribute to finding further fundamental properties and potential exploitation of the self-assembled CNF-based composite materials.

## 2. Fabrication of Self-Assembled Chitin Nanofibers upon the Bottom-Up Approach

**Figure 3:**
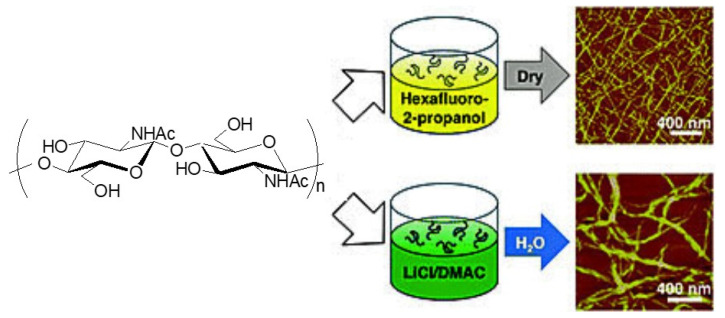
A schematic of the chitin nanofiber preparation routes (reproduced and adapted with permission from reference [28], Copyright 2010, Royal Society of Chemistry.

The self-assembled ChNFs were prepared by regeneration from the solutions of chitin [28]. Hexafluoro-2-propanol (HFIP) and LiCl/*N, N*-dimethylacetamide (DMAc) were used as the solvents for this process [4]. The self-assembling process successfully progressed either via solvent evaporation for the HFIP system or via the addition of water for the LiCl/DMAc system (Figure 3). The self-assembling process from the solution of chitin in HFIP was simply conducted, in which slowly drying the solutions of appropriate concentrations led to long (10–100 μm) nanofibers with a small diameter (2.8 ± 0.7 nm). Owing to the low volatility of LiCl/DMAc, on the other hand, such a drying process was not applicable. Alternatively, the addition of ample amounts of water into the solution of chitin in LiCl/DMAc resulted in precipitation of chitin, which had nanofiber morphology with a larger diameter (10.2 ± 2.9 nm) than those prepared from HFIP, but similar length as above. The above mild conditions during regeneration remarkably did not cause deacetylation of acetamido groups and depolymerization of chitin chains.

Structure-properties-processing relationships were developed to fabricate flat and high-quality chitin nanofiber films from the self-assembled ChNFs, forged from the chitin/HFIP solution, by different methods (cold-press, vacuum drying, and vacuum-assisted filtration) [29]. Processing method, drying time, and solution concentration affected and provided control over nanofiber film density, network structure, and mechanical properties. Free-standing micropatterned substrates were also obtained from the self-assembled ChNFs [30]. The resulting ultrathin micropatterned substrates were mechanically robust yet flexible and easy to manipulate. A paper-like ChNF sheet was fabricated—using a centrifugal casting technique from the chitin/HFIP solution—into a large-area uniform sheet that exhibited good paper properties (foldability and printability), an optical transmittance of ≈92%, and an elastic modulus of 4.3 GPa [31].

**Figure 4:**
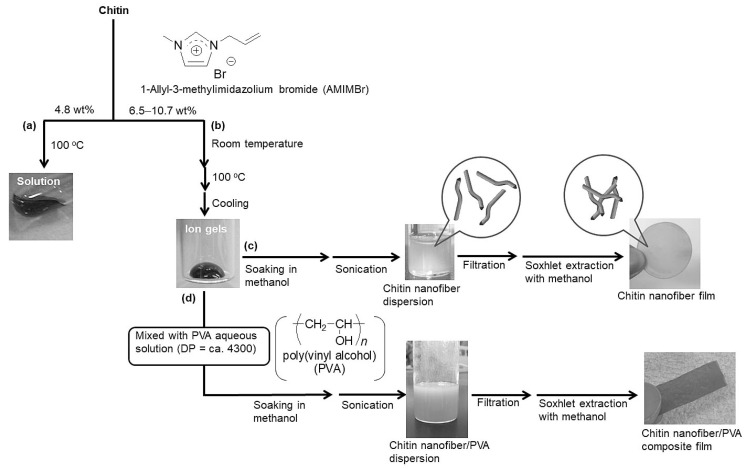
(**a**) Dissolution and (**b**) gelation of chitin with AMIMBr and preparation of (**c**) self-assembled ChNF dispersion/film and (**d**) ChNF/PVA composite film (reprinted with permission from ref. [27]. Copyright 2020, Elsevier).

Ionic liquids (ILs) have been used as the media in preparing ChNFs via a regenerative self-assembling approach [26,27]. ILs are molten salts melted at temperatures below the boiling point of water. They have been identified as quality solvents for cellulose [32,33,34,35,36,37] since Rogers et al. have found the dissolution of cellulose in an ionic liquid, 1-butyl-3-methylimidazolium chloride (BMIMCl) [38]. Separate from the cellulose dissolution, ILs for the dissolution of chitin were rarely found until almost one-and-a-half decade ago [39,40,41,42,43]. A first study for the dissolution of chitin in ILs was reported in 2008, which was achieved using 1-butyl-3-methylimidazolium acetate [44]. In 2009, another ionic liquid, 1-ally-3-methylimidazolium bromide (AMIMBr), was found to dissolve chitin in concentrations up to 4.8 wt% by heating at 100 °C (Figure 4a) [45]. Moreover, it formed gel-like materials with chitin in higher concentrations (6.5–10.7 wt%) by standing mixtures at room temperature, followed by heating at 100 °C (Figure 4b). The dissolution of chitin with deep eutectic solvents (DESs), as ionic liquid analogs, composed of mixtures of choline halide-urea, chlorocholine chloride-urea, and choline chloride-thiourea were also reported [46,47]. DESs are fluids formed by the adequate mixtures of the hydrogen bond acceptors and donors, which are capable of self-association through hydrogen bonding interactions to form eutectics with lower melting points in comparison to each individual component. DESs, prepared from imidazolium ionic liquids having different substituents, e.g., 1-butyl- and 1-ethyl-3-methylimidazolium bromides and chlorides, as well as thiourea, were also found to dissolve chitin (~5 wt%) [48].

From the 6.5–10.7 wt% chitin ion gels with AMIMBr, ChNF dispersions were obtained by immersion in methanol at room temperature for 24 h for the slow regeneration of chitin, followed by sonication (Figure 4c) [49]. The SEM image of the sample, which was prepared by dilution of the resulting dispersion with methanol, showed the nanofiber morphology with ca. 20–60 nm diameters and several hundred lengths, indicating the self-assembling formation of ChNFs upon the bottom-up process from the ion gels. When the resulting self-assembled ChNFs were isolated by filtration of the dispersion, a ChNF film was fabricated. The SEM image of the resulting film observed the morphology of highly entangled nanofibers, in which such an entangled structure from ChNFs probably contributed to the formation of the film. The regeneration from the chitin ion gels with AMIMBr using calcium halide·2H_2_O/methanol solutions affected the morphologies of self-assembled chitin nanofibers, in which the self-assembled chitin nanofibers with a higher aspect ratio were fabricated by the regeneration using CaBr_2_·2H_2_O/methanol solution in lower concentration [50].

**Figure 5:**
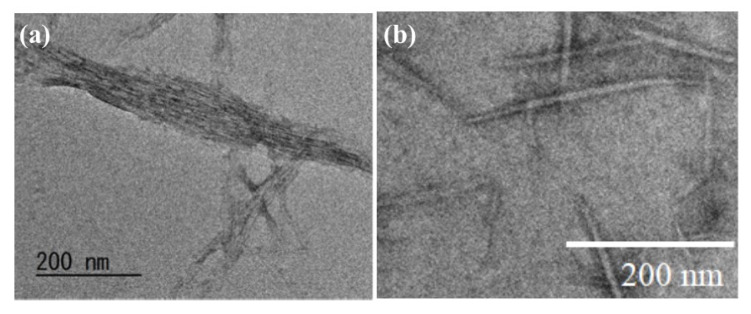
TEM images of: (**a**) self-assembled ChNFs; and (**b**) SD-ChNFs.

Moreover, in the following study, the TEM image of the ChNF dispersion supported that the self-assembled ChNFs were constructed by bundles upon hierarchically assembling from thin fibrils (Figure 5a) [51]. The self-assembled ChNF film was treated with aqueous NaOH for partial deacetylation of acetamido groups and subsequently disintegrated by cationization and electrostatic repulsion in 1.0 mol/L aqueous acetic acid, with ultrasonication giving a dispersion of thinner nanofibers named ‘scaled-down (SD)-ChNFs’ [52]. Disintegration of the bindles was confirmed by the TEM image of the resulting dispersion (Figure 5b). Isolation of the SD-ChNFs via filtration of the dispersion resulted in a highly flexible film that bent and twisted with ease.

The self-assembled ChNFs were also formed by gelation of chitin with the choline chloride-thiourea DES system (10% *w*/*w*), followed by dilution with water [53]. Furthermore, a paper-like chitin sheet comprising longer ChNFs, named ‘chitin paper’, was fabricated via regeneration from the solution of chitin in DES of 1-allyl-3-methylimidazolium/thiourea [54]. The chitin paper exhibited better mechanical properties than the abovementioned self-assembled ChNF film.

## 3. Preparation of Composite Materials from Self-Assembled Chitin Nanofibers

The self-assembled ChNFs, fabricated from the solutions in HFIP, were employed as a biomimetic extracellular matrix for the attachment of primary neurons in vivo. Surfaces on ChNFs were deacetylated to form 4 nm and 12 nm diameter chitosan nanofibers that were further composited with poly(D-lysine) to examine combinatory effects and structurally analyzed by atomic force microscopy [55]. The combination promoted neuron attachment, neurite coverage, and cell survival compared with ChNFs alone.

A ChNF-silk biocomposite was facilely co-assembled from the homogeneous chitin (extracted from squid pen)-silk (fibroin extracted from *B. Mori* cocoon)/HFIP solutions [56]. ChNFs self-assembled inside the silk matrix to fabricate the biocomposite that mimicked the organic phase of the insect cuticle and the exoskeleton of crustaceans, which were made of ChNFs embedded in a silk-like protein matrix. The biocomposite was structured by strong hydrogen bonding between ChNFs and the surrounding silk matrix, resulting in an increase in elastic modulus.

**Figure 6:**
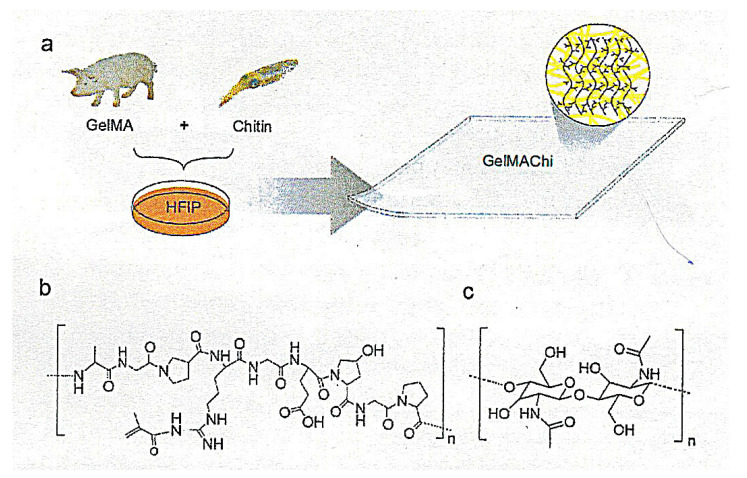
(**a**) Schematic illustration for the self-assembling process of the biomimetic ChNF-GelMA films; (**b**) molecular structure of GelMA; and (**c**) molecular structure of chitin (reprinted with permission from ref. [57]. Copyright 2016, Royal Society of Chemistry).

The co-assembling approach was also applied to fabricating ultrastrong and flexible hydrogels from ChNFs and gelatin methacryloyl (GelMA) (Figure 6) [57]. To forge the GelMA-chitin hydrogels, chitin and GelMA, co-dissolved in HFIP, were first dried on a polydimethylsiloxane mold to form films. Exposure of the films to UV light at 365 nm for 3 min in the presence of a photoinitiator led to the crosslinking of the methacryloyl groups in GelMA, producing a covalently bonded matrix intertwined with ChNFs. The ChNF reinforcement increased the hydrogel elastic modulus by one-thousand-fold and train-to failure by >200% improving handling and integrity for tissue engineering applications.

The self-assembled ChNFs, obtained from the ion gel with AMIMBr, were composited with poly(vinyl alcohol) (PVA) by a co-regeneration process to fabricate the ChNF/PVA composite material (Figure 4d) [49]. After a solution of PVA (DP = ca. 4300) in a small amount of hot water was mixed with the 9.1 wt% chitin ion gel with AMIMBr, the co-regeneration of the two polymeric components progressed by immersing the mixture in methanol as a poor solvent for both the polymers. The subsequent filtration and Soxhlet extraction with methanol resulted in the self-assembled ChNF/PVA composite film. The SEM image of the composite film observed that the nanofiber morphology remained and PVA components filled in spaces among ChNFs. The DSC profile of the composite film exhibited an endothermic peak assignable to a melting point of PVA, suggesting that PVA in the composite film formed a crystalline structure owing to the relative immiscibility of chitin and PVA. However, the melting point peaks from PVA shifted to lower temperatures accompanied by broadening when the contents of chitin in the composite films increased, indicating that chitin and PVA might be partly miscible at the interfacial area between the two polymeric components in the composite films by the formation of hydrogen bonding between them. Furthermore, the tensile testing of the composite films showed the enhancement of the mechanical properties by increasing the ratios of PVA to chitin.

The self-assembled ChNFs were used as stabilizers for the Pickering emulsion polymerization of styrene [58]. Pickering emulsions are those of any type, either oil-in-water, water-in-oil, or even multiple, stabilized by solid particles or other types of solid materials in place of surfactants in general emulsions. Prior to the emulsion polymerization, anionic carboxylate groups were introduced to ChNFs by reaction with maleic anhydride in the presence of perchloric acid, which were dispersed well in aqueous ammonia. Radical polymerization by potassium persulfate as an initiator was then conducted at 70 °C in the emulsion, in which styrene droplets were stably surrounded by ChNFs in aqueous ammonia to fabricate the composite particles. The particle sizes were changed depending on ChNF/styrene feed ratios. Furthermore, the composite particles were facilely converted into the ChNF-based hollow particles by solubilizing out the styrene core with toluene. However, isolating the hollow particles from the toluene dispersion and redispersing them in water was attempted, the SEM image of a spin-coated sample from the aqueous dispersion warned against retaining the hollow morphology. This observation indicated instability of the shells in the hollow structure against the above isolation and redispersion procedures, which were formed by the physical interaction of polystyrene with ChNFs. When the abovementioned SD-ChNFs were used as stabilizers for the Pickering emulsion polymerization of styrene, smaller composite particles than those using the self-assembled ChNFs were produced [59].

**Figure 7:**
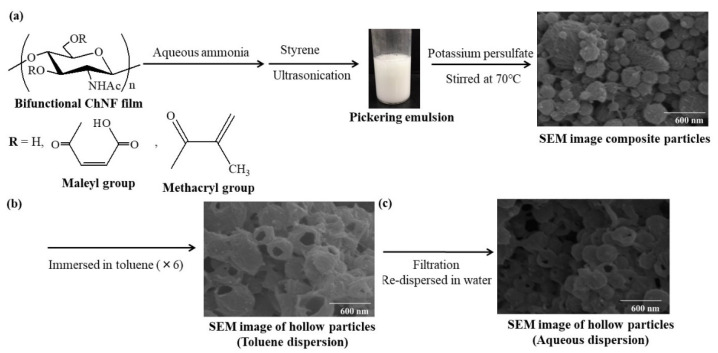
(**a**) Preparation of ChNF/polystyrene composite particles by Pickering emulsion polymerization; (**b**) conversion into hollow particles by treatment with toluene; and (**c**) redispersion of hollow particles in water.

In addition to anionic maleyloyl groups on ChNFs, polymerizable methacryloyl groups were also substituted as a second functionalization to provide the ability in copolymerization with styrene for stabilization of the hollow structure [60]. After the formation of styrene-in-water (aqueous ammonia) Pickering emulsion using the resulting bifunctional ChNFs as stabilizers, radical polymerization progressed by potassium persulfate at 70 °C to obtain the composite particles (Figure 7a). The hollow particles were then formed by solubilizing out inner polystyrene with toluene (Figure 7b), which stably redispersed in water (Figure 7c). A fluorescent dye, pyrene, was encapsulated into the cavity of the hollow particles by hydrophobic interaction with polystyrene present on the inner walls. The dye was ready to be released following treatment of the resulting fluorescent hollow particles with surfactant and oleyl alcohol in water.

**Figure 8:**
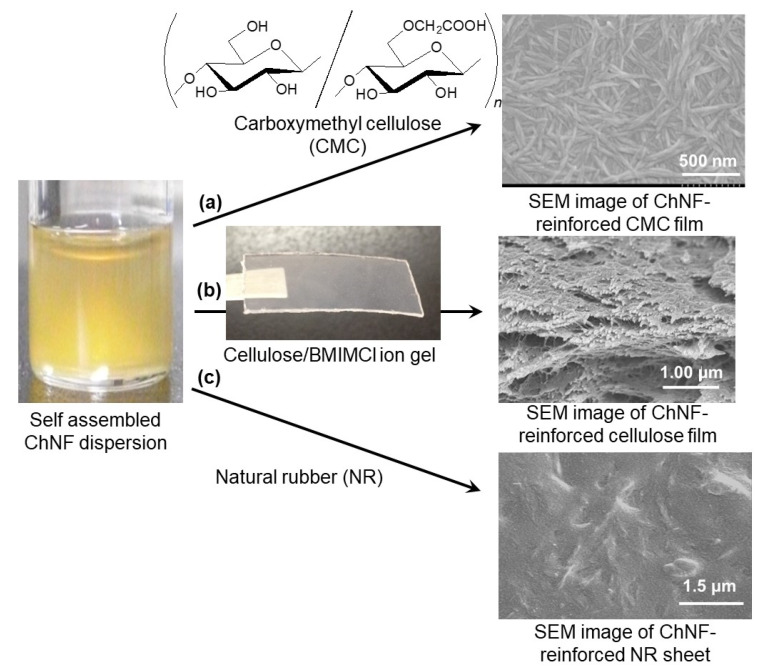
Preparations of ChNF-reinforced: (**a**) CMC film; (**b**) cellulose film; and (**c**) NR sheet.

The self-assembled ChNFs have been used as a reinforcing agent through composition with other polymers. Since chitin was regarded a cationic polysaccharide because of the presence of several percent of free amino groups in total repeating units by deacetylation of acetamido groups, the self-assembled ChNFs were used as a reinforcing agent for an anionic polysaccharide, that is, carboxymethyl cellulose (CMC), by electrostatic interaction [61]. CMC films, prepared by casting technique, were immersed in the self-assembled ChNF/methanol dispersions with the different contents, followed by centrifugation and drying, to fabricate the ChNF-reinforced films (Figure 8a). The SEM image of the resulting film observed the presence of nanofibers on the surface.

The self-assembled ChNF-reinforced cellulose films were also fabricated [62]. An ionic liquid, BMIMCl, was found to form an ion gel with cellulose [63]. Therefore, when the cellulose/BMIMCl ion gels were immersed in the self-assembled ChNF/methanol dispersions with varying contents, two polysaccharides were composited through regeneration of cellulose to fabricate the ChNF-reinforced cellulose films (Figure 8b). The unit ratios of ChNFs to cellulose in the films increased in accordance with ChNFs contents in the methanol dispersions. The SEM image of the cross-sectional area in the resulting film showed the tips of ChNFs extending from the solid, indicating that ChNFs were present not only on the surface but also inside the film. The amounts of ChNFs in the CMC and cellulose composite films strongly affected the enhancement of the mechanical properties under a tensile mode, supporting their reinforcing effect.

Fabrication of the self-assembled ChNF-reinforced natural rubber (NR) sheet was investigated [64]. The self-assembled ChNF dispersion with aqueous ammonia was mixed with NR latex stabilized with aqueous ammonia, followed by drying under reduced pressure to obtain the ChNF-reinforced NR sheet (Figure 8c). The SEM image of the resulting sheet observed the independent ChNF morphology in the area of NR solid, suggesting that ChNFs were dispersed well in the NR latex. The tensile testing of the sheet indicated the reinforcing effect of ChNFs in the sheet. When the above ChNF/NR dispersions with aqueous ammonia were heated to evaporate ammonia, followed by lyophilization, a porous material was obtained. By evaporating the ammonia stabilizer, the nanofibers were aggregated with NR, which were then agglomerated to form spaces between them, resulting in porous morphology.

A composite film of the abovementioned SD-ChNFs with *ι*-carrageenan, a sulfated anionic polysaccharide, was produced via multi-point ionic cross-linking [52]. The stress-strain curve of the SD-ChNF/*ι*-carrageenan composite film under tensile mode exhibited an enhanced elongation at break and a tensile strength comparable to that of the SD-ChNF film. These enhanced mechanical properties and efficient compositing properties were attributed to the scaling down of the ChNFs.

## 4. Conclusions and Outlook

Based on a regenerative bottom-up approach for the fabrication of ChNFs, in this short review article, the preparation of ChNF-based composite materials with the different polymeric components has been overviewed. Several practical forms, such as films, sheets, scaffolds, and particles, were obtained by efficient compositions from self-assembled ChNFs and other polymeric substrates. As the self-assembled ChNFs show considerable advantages in various application fields, such as food, agricultural, and biomedical industries, the studies on the fabrication of ChNF-based composite materials upon the bottom-up approach have been significantly developed in the last one-and-a-half decade and will increasingly attract much attention in the application fields related to biomedical and environmental industries in the future. In particular, the regenerative bottom-up process can be facilely conducted without use of special equipment, which is advantageous compared with the top-down process. Based on these viewpoints, therefore, further investigations in developing new regenerative preparation methods for the self-assembled ChNFs with different morphologies and sizes will be attempted in laboratory and bench scales to find new properties and applications. New solvent systems will be also developed for the cost-effective fabrication of ChNFs upon the bottom-up process.

## Data Availability

Not applicable.
